# A case study of impacts of an extreme weather system on the Mediterranean Sea circulation features: Medicane Apollo (2021)

**DOI:** 10.1038/s41598-023-29942-w

**Published:** 2023-03-08

**Authors:** Milena Menna, Riccardo Martellucci, Marco Reale, Gianpiero Cossarini, Stefano Salon, Giulio Notarstefano, Elena Mauri, Pierre-Marie Poulain, Antonella Gallo, Cosimo Solidoro

**Affiliations:** Oceanographic Section, National Institute of Oceanography and Applied Geophysics - OGS, 34010 Sgonico (TS), Italy

**Keywords:** Biogeochemistry, Environmental sciences, Natural hazards, Ocean sciences

## Abstract

The attention of the scientific community, policymakers, and public opinion on the Medicanes has recently grown because of their increase in intensity and harmful potential. Although Medicanes may be influenced by pre-existing upper-ocean conditions, uncertainties remain about how such weather extremes influence ocean circulation. This work examines a condition that has been never described before in the Mediterranean, which involves the interplay between an atmospheric cyclone (Medicane Apollo—October 2021) and a cyclonic gyre located in the western Ionian Sea. During the event, the temperature in the core of the cold gyre dropped dramatically, due to a local maximum in the wind-stress curl, Ekman pumping, and relative vorticity. Cooling and vertical mixing of the surface layer combined with upwelling in the subsurface layer caused a shoaling of the Mixed Layer Depth, halocline, and nutricline. The resulting biogeochemical impacts included an increase in oxygen solubility, chlorophyll concentration, productivity at the surface, and decreases in the subsurface layer. The presence of a cold gyre along Apollo's trajectory leads to a different ocean response from that observed with previous Medicanes, endorsing the efficiency of a multi-platform observation system integrated into an operational model for future mitigation of weather-related damages.

## Introduction

Severe weather systems are important components of the global atmospheric circulation and play a fundamental role in shaping the water budget, the location and the magnitude of extreme events such as heavy rains, windstorms, marine storminess, storm surges, and landslides^1–5^. In particular, tropical cyclones (TCs) are intense weather systems that originate over tropical oceans. They are among the most significant threats to human life and property in the world^6–8^, damaging offshore platforms, threatening coastal areas, and causing economic and human losses^9,10^.

TCs are generally associated with strong air-sea interactions and vigorous responses in the upper layer of the ocean^11^, whose strength depends on the characteristics of the TCs themselves (e.g. intensity, wind speed, and size) and on the pre-existing ocean conditions (e.g. stratification and presence of eddies). The upper ocean response typically consists of: (1) sea surface cooling and subsurface warming (“heat pump” effect;^12,13^), driven by a combination of different physical processes (Ekman pumping, geostrophic advection, and ocean vertical mixing;^12,14^); (2) strong vertical mixing, upwelling, near-inertial waves, and currents, induced by the transfer of mechanical energy from the atmosphere to the ocean^8,15–19^; (3) enhancement of phytoplankton productivity i.e. upward mixing of nutrients and redistribution of the deep chlorophyll-a maximum (DCM) throughout the mixed layer^20,21^.

The Mediterranean region is one of the areas on the global scale where severe weather system activity is more frequent^22–24^. Although the environmental conditions do not allow the development of true TCs^24^, some mid-latitude cyclones crossing the Mediterranean Sea occasionally undergo a transition giving rise to weather systems similar to TCs^25,26^. These weather systems, known as Mediterranean Hurricanes or Medicanes, are smaller than their tropical counterparts^27–29^. They are characterized by a peculiar cyclonic axisymmetric structure, cloud bands wrapped around a low-pressure and warm-windless core surrounded by strong winds^26,30^, and are associated with extreme weather phenomena^24,26,31–33^. In recent years, the scientific community's attention on Medicanes is growing because of their large harmful potential on the land and coastal areas^24,26,34,35^, calling for coordinated and interdisciplinary research efforts^24^. In this context, the oceanographic community should address some outstanding aspects of the air-sea interaction during the events, assess the Medicanes’ impacts on the marine environment dynamics, and support operational oceanography in predicting these extreme events. Studies on the oceanographic response to Medicanes started only recently, thanks to the works carried out by^35–37^, who focused on the Medicanes Qendresa (2014), Zorbas (2018) and Ianos (2020), respectively. The case history is therefore still limited, and the interaction between Medicanes and dynamic structures (eddies or gyres) in the Mediterranean still calls for investigations to fully understand the extent of impacts on marine dynamics. These interactions can modulate the intensity and the harmful potential of atmospheric systems themselves and significantly influence the mass and heat transports in the ocean, eventually having a critical cumulative effect on the climate^38,39^.

**Figure 1:**
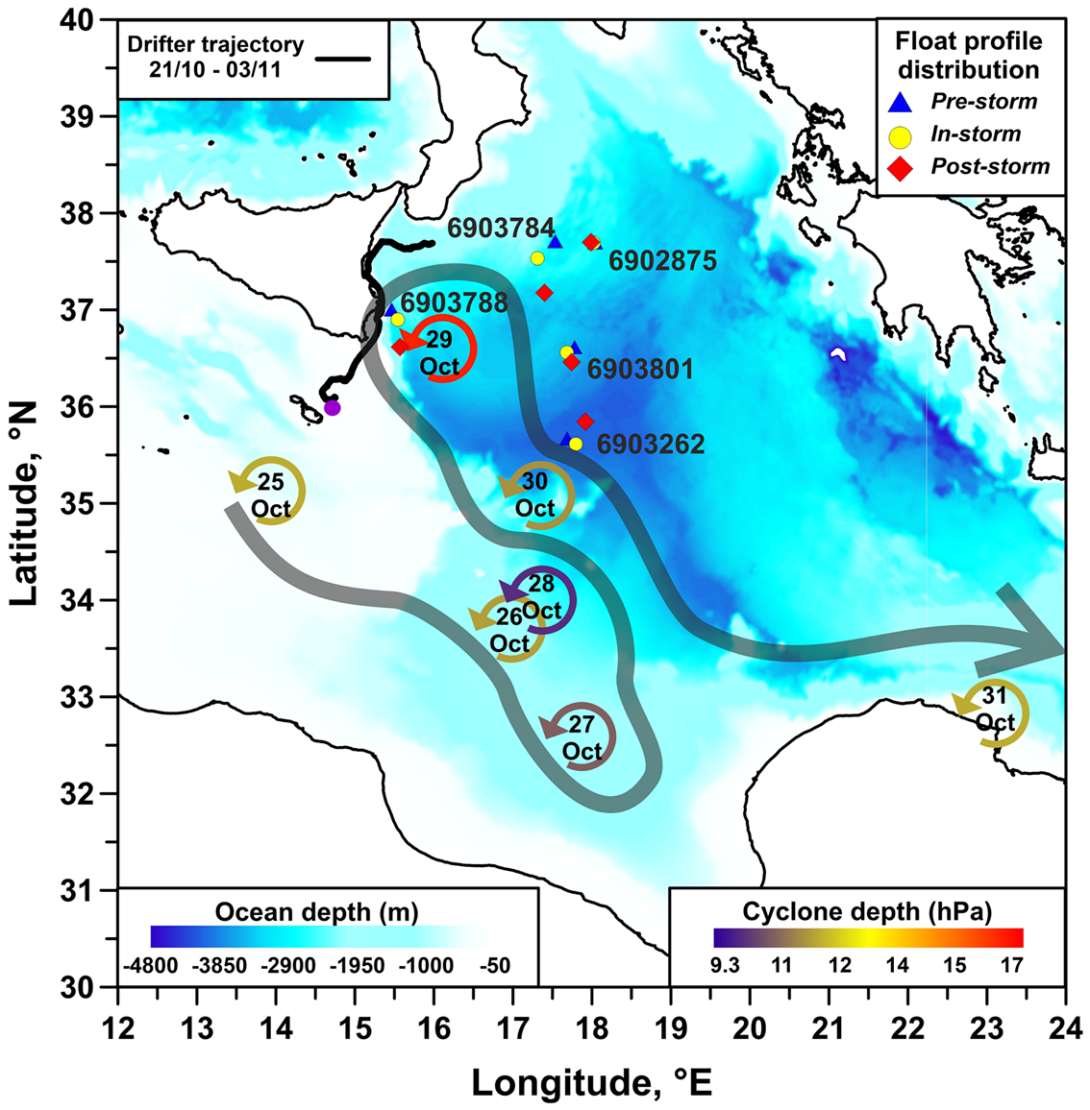
Argo float positions (colored symbols and identification numbers) and track of the drifter IMEI 300234067977120 (black line) superimposed on the geography and bathymetry of the Ionian Sea (sea bottom depth in blue shade). Circular arrows indicate the daily position of the Medicane Apollo from 25 October 2021 12:00 UTC to 31 October 2021 12.00 UTC; arrows are colored by cyclone depth. The gray shaded large arrow gives a schematic view of the path carried out by the Medicane Apollo.

**Figure 2:**
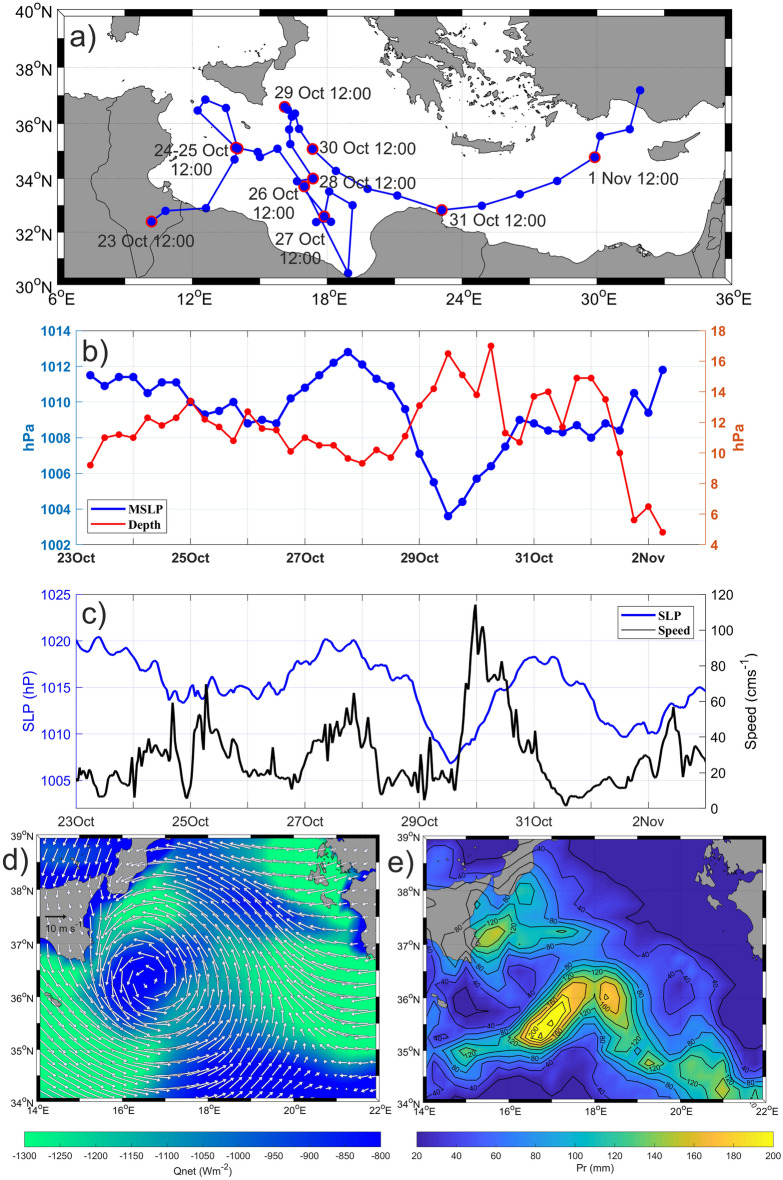
(**a**) 6 h Medicane Apollo track between 23 October 2021 12:00 UTC to 2 November 2021 06:00 UTC (blue dots); Medicane daily positions at 12:00 UTC are circled in red. Time series of (**b**) MSLP and cyclonic depth (i.e. a metric for the cyclone intensity) derived from ERA5, (**c**) SLP and current speed derived from drifter IMEI 300234067977120. Cumulative heat losses (**d**) and precipitation (**e**) in the period 27–30 October 2021.

In October 2021, the Sicily Channel and the central-western Ionian Sea were affected by the passage of the Medicane Apollo (Figs. 1, 2a) that caused intense precipitations, huge coastal floodings, seven fatalities, and large damages in Sicily and Calabria. During its lifetime, the system crossed an area of the Ionian Sea characterized by the presence of a cyclonic ocean vortex offering, for the first time in the Mediterranean Sea, the chance to describe the impact of a Medicane on a pre-existing cold circulation feature. During the event, the Ionian Sea was monitored by autonomous instruments (drifter and floats; Fig. 1), as well as satellite and model products. We used these data to investigate the physical and biogeochemical responses of the oceans to the impact of Medicane Apollo.

## Results

This section describes the temporal evolution of Apollo and its impacts on the oceanographic (physical and biogeochemical) features and the weather variables in the area crossed by the system. Weather variables were derived from ERA5, the fifth generation of the ECMWF reanalysis^40^; satellite data^41,42^, and model products (^35,42^; hereafter CM model) were distributed by the Copernicus Marine Service; in-situ float and drifter data were obtained from Argo website^43,44^ and from the OGS drifter database^45^, respectively (for more details see Methods Section).

The event was studied by delimiting the ocean area impacted by the system and considering three time windows, each lasting four days, corresponding to the conditions before (hereafter “Pre-storm”, 21–24 October 2021), during (hereafter “In-storm”, 27–30 October 2021), and after the passage of the system (hereafter “Post-storm”, 31 October 2021–3 November 2021). The quantitative analysis in each time window and for each physical/biogeochemical variable, as well as the comparisons between in-situ and model-derived variables, are summarized in Table 1.

**Table 1:** Mean distribution of the Ekman pumping, Ekman depth, Ocean Heat Content (OHC), temperature (T), salinity (S), Absolute Dynamic Topography (ADT), Significant Wave Heigh (SWH), and chlorophyll-a (Chl) in the Center and in the Edge sectors of the ocean area impacted by the Medicane Apollo, during the Pre-storm, In-storm, Post-storm time periods. The last column shows the difference between the Post-storm and Pre-storm conditions for each parameter (∆). Mean values of OHC, T, and S defined by the CM model in the Edge sector are compared with those obtained from the floats located in the same sector. Mean values of Chl in the surface layer are compared with that obtained from the satellite in the Center and Edge sectors.

		Pre-storm	In-storm	Post-storm	
CENTER	**Ekman pumping** (10^–5^ m s^−1^)	− 0.06	2.5	− 0.2	
	**Ekman depth **(m)	50.8	87.1	56.7	
	**OHC** (10^10^ J m^−2^) 0–150 m				**∆OHC** (10^10^ J m^−2^)
	CM model	1.13	1.10	1.07	− 0.06
	**T** (°C) 0–150 m				**∆T** (°C)
	CM model	18.01	17.55	17.07	− 0.94
	**S**				**∆S**
	CM model 0-MLD	38.56	38.50	38.54	− 0.02
	CM model MLD-150 m	38.74	38.83	38.90	0.16
	**ADT** (cm)	− 4.29	− 3.70	− 3.64	**∆ADT **0.65
	**SWH** (m)	1.20	3.21	1.29	**∆SWH **0.09
	**Chl **(mg m^−3^)				**∆Chl** (mg m^−3^)
	CM model 0-MLD	0.06	0.08	0.08	0.02
	CM model MLD-150 m	0.32	0.26	0.18	− 0.14
	Satellite	0.06	0.09	0.11	0.05
EDGE	**Ekman pumping** (10^–5^ m s^−1^)	− 0.16	1.9	− 0.21	
	**Ekman depth** (m)	56.7	108.1	57.4	
	**OHC** (10^10^ J m^−2^) 0–150 m				**∆OHC** (10^10^ J m^−2^)
	CM model	1.15	1.13	1.11	− 0.04
	float 6903801	1.07	1.05	1.07	0
	float 6903788	1.15	1.08	1.01	− 0.14
	float 6903262	1.15	1.13	1.10	− 0.05
	**T** (°C) 0–150 m				**∆T** (°C)
	CM model	18.31	17.99	17.69	− 0.62
	float 6903801	18.01	17.65	17.92	− 0.09
	float 6903788	19.26	18.18	17.25	− 2.01
	float 6903262	19.29	18.92	18.59	− 0.7
	**S 0-MLD**				**∆S**
	CM model	38.54	38.46	38.41	− 0.13
	float 6903801	38.03	38.08	37.92	− 0.11
	float 6903788	38.96	38.78	38.44	− 0.52
	float 6903262	38.12	38.03	37.95	− 0.17
	**S MLD-150 m**				**∆S**
	CM model	38.54	38.57	38.61	0.07
	float 6903801	38.51	38.71	38.67	0.16
	float 6903788	38.76	38.80	38.84	0.08
	float 6903262	38.19	38.23	38.21	0.02
	**ADT** (cm)	4.43	4.6	5.45	**∆ADT** 1.02
	**SWH** (m)	1.23	3.25	1.25	**ΔSWH** 0.02
	**Chl **(mg m^−3^)				**∆Chl** (mg m^−3^)
	CM model 0-MLD	0.05	0.06	0.07	0.02
	Model MLD-150 m	0.24	0.21	0.19	− 0.05
	Satellite	0.05	0.08	0.09	0.04

### The temporal evolution of Medicane Apollo and related weather variables

The trajectory of the Medicane Apollo was reconstructed by applying an objective procedure based on searching the minimum in the 6-hourly 0.25° ERA5 Mean Sea Level Pressure (MSLP) fields (Fig. 2a;^46^). According to the procedure, this storm started its life cycle as a relatively shallow system near the Tunisian coast (31.50° N, 8.78° E) on October 23^rd^. From there, it moved towards an area between the Sicily Channel and the southern Ionian Sea where it was stuck until October 31^st^ when, after a quick passage over the Levantine, it dissipated on the Turkish coast between 1 and 2 November (Fig. 2a).

The temporal evolution of the Apollo pressure field shows that the system reached its maximum intensity on October 29^th^, when it brushed the southeastern Sicilian coast (Fig. 2a,b), exhibiting the maximum cyclone depth (defined as a metric for the cyclone intensity that takes into account both MSLP at the center of the system and in the surrounding environment;^47^) and the lowest MSLP (Figs. 1, 2b). The time series of Sea Level Pressure (SLP) recorded by the drifter Surface Velocity Program Barometer (SVPB; code IMEI 300234067977120), confirms the temporal evolution of the SLP minima at sea surface on October 29^th^ (Figs. 1, 2c). Simultaneously, the surface current field dramatically strengthened, with speeds rising from ~ 15 to 118 cm s^−1^ in a few hours (Fig. 2c), in a wind-speeds regime of 6–9 m·s^-1^ along the eastern Sicilian coast (not shown). These timeseries confirms the coupling between the ocean and the atmosphere during Apollo.

During the “In-storm” period, the weather system affected the whole central Ionian Sea with a maximum wind speed of 12 m s^−1^ and larger cumulative heat losses (as large as − 1200 W m^−2^) mainly located along its border (Fig. 2d). Cumulative heat losses exceeded the “Pre-storm” and “Post-storm” conditions by approximately 400–800 W m^−2^ (not shown). Cumulative precipitations (Fig. 2e) accounted for larger values in the southeastern branch of the cyclone (exceeding the “Pre-storm” and “Post-storm” conditions by ~ 100–160 mm) and along the southeastern Sicilian coast (exceeding the “Pre-storm” and “Post-storm” conditions by ~ 60–120 mm). The latter is the area where the most severe coastal damages occurred.

### Marine environment conditions in the ocean-impacted area

**Figure 3:**
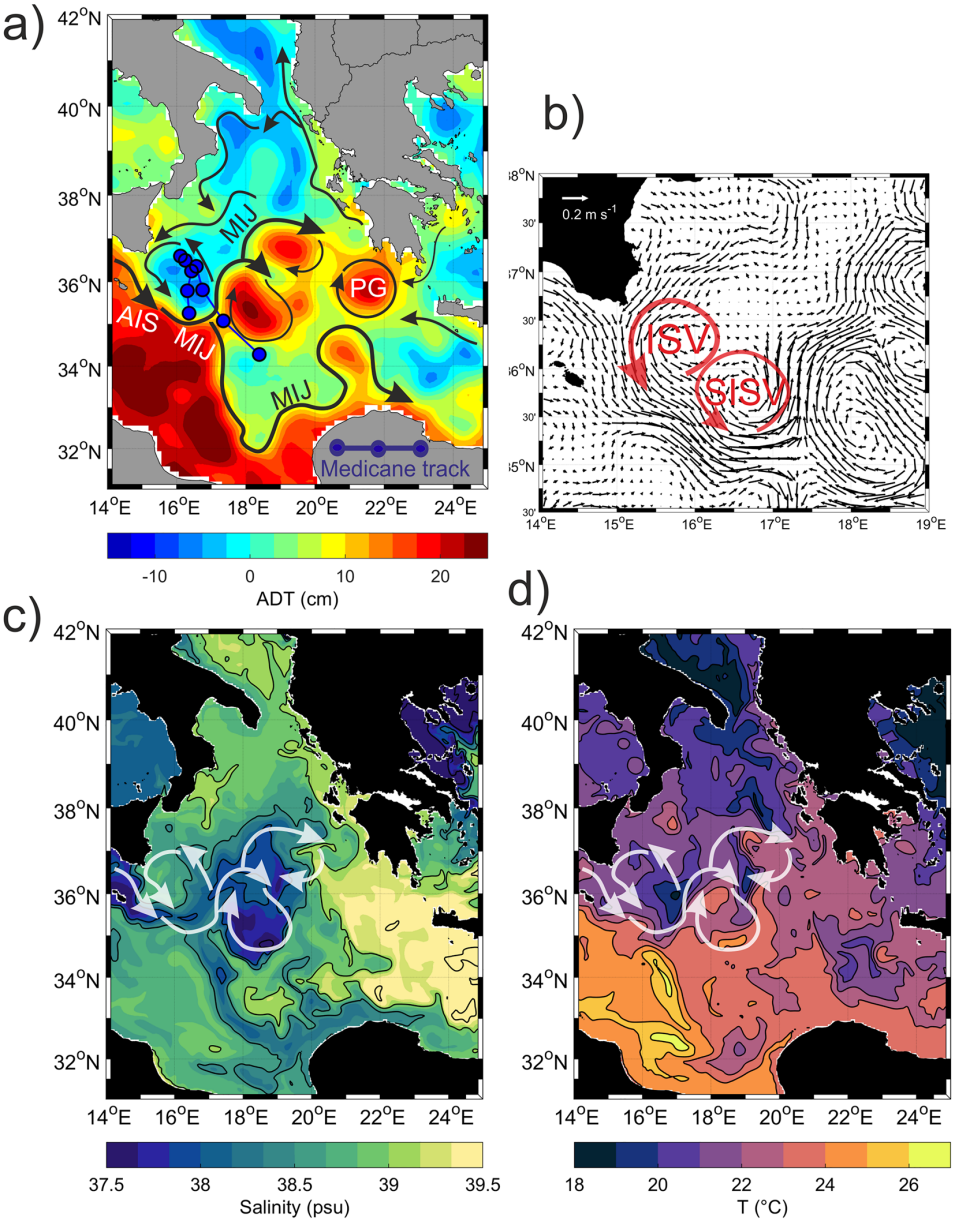
Oceanographic conditions on October 29^th^, 2021: (**a**) Schematic representation of the main currents and circulation structures (black arrows) superimposed on the ADT field (colors); 6 h Medicane Apollo track in the period 28 October 2021 18:00 UTC—30 October 2021 18:00 UTC (blue line and dots); (**b**) zoom on the absolute geostrophic velocities in the Medicane impacted area; (**c**) salinity and (**d**) temperature fields derived from the physical CM model in the surface layer (0–10 m depth); the main currents are superimposed with white arrows. Acronyms: AIS—Atlantic Ionian Stream; MIJ—Mid Ionian Jet; PG—Pelops Gyre; ISV—Ionian Shelf break Vortex; SISV—Southern Ionian Shelf break Vortex.

On 29 October, a weak cyclone was present in the north-western sector of the Ionian Sea while intense jet currents, sub-basin scale anticyclones, and mesoscale vortexes occurred in its south-eastern and south-western sectors (Fig. 3a,b, see also below). Relative cold and fresh Atlantic Water (AW) spread in the Ionian Sea following the Atlantic-Ionian Stream (AIS) and the Mid-Ionian Jet (MIJ) pathways (Fig. 3a,c,d). North and east of the MIJ signature, the Ionian was filled by relative warmer and saltier waters of Levantine origin (Levantine Surface Waters—LSW; Fig. 3c,d).

The MIJ showed a pronounced meandering behavior, related to strong temperature and salinity gradients. East of 16°E, it split into two branches (Fig. 3a): a meridional, southward branch that transported AW toward the African coast and then joined the eastward coastal current; a northeastward branch that fed two intense anticyclonic vortexes in the central Ionian (35°–37.5° N; 18°–20° E). Two mesoscale cyclonic permanent structures, the Ionian Shelf break Vortex (ISV) and the Southern Ionian Shelf break Vortex (SISV) were located between the eastern Sicilian coast and the open Ionian Sea (Fig. 3a,b), the same area crossed by the Medicane (Fig. 3a). The ISV is a wind-driven structure, generally more intense in winter, triggered by the wind-stress curl along the eastern coast of Sicily^48^. On the other hand, the SISV dynamics is driven by the interaction between surface currents and topography and its intensity is modulated by the large-scale internal variability of the Northern Ionian^48,49^. A cyclonic circuit, flanked to the southeast by the MIJ, characterized the whole area surrounding these two dynamic structures (Fig. 3b), that were merged together forming a two-lobe sub-basin scale structure, defined hereafter as “cyclonic gyre”. Surface temperatures ranged from 20 to 23 °C and from 23 to 26 °C in the northern and southern Ionian, respectively (Fig. 3d) with the largest values observed along the African coast. It is interesting to note that the core of the SISV was filled with waters colder than its surrounding areas, suggesting an intense upwelling activity in its interior. The Medicane-impacted region was characterized by the presence of a strong thermohaline front between the warmer (~ 23 °C) and less saline (37.6–38.2) AW advected by the MIJ, and the colder (19–21 °C) and more saline (38.4–38.8) Ionian waters, recirculating in the region of ISV and SISV (Fig. 3b–d).

**Figure 4:**
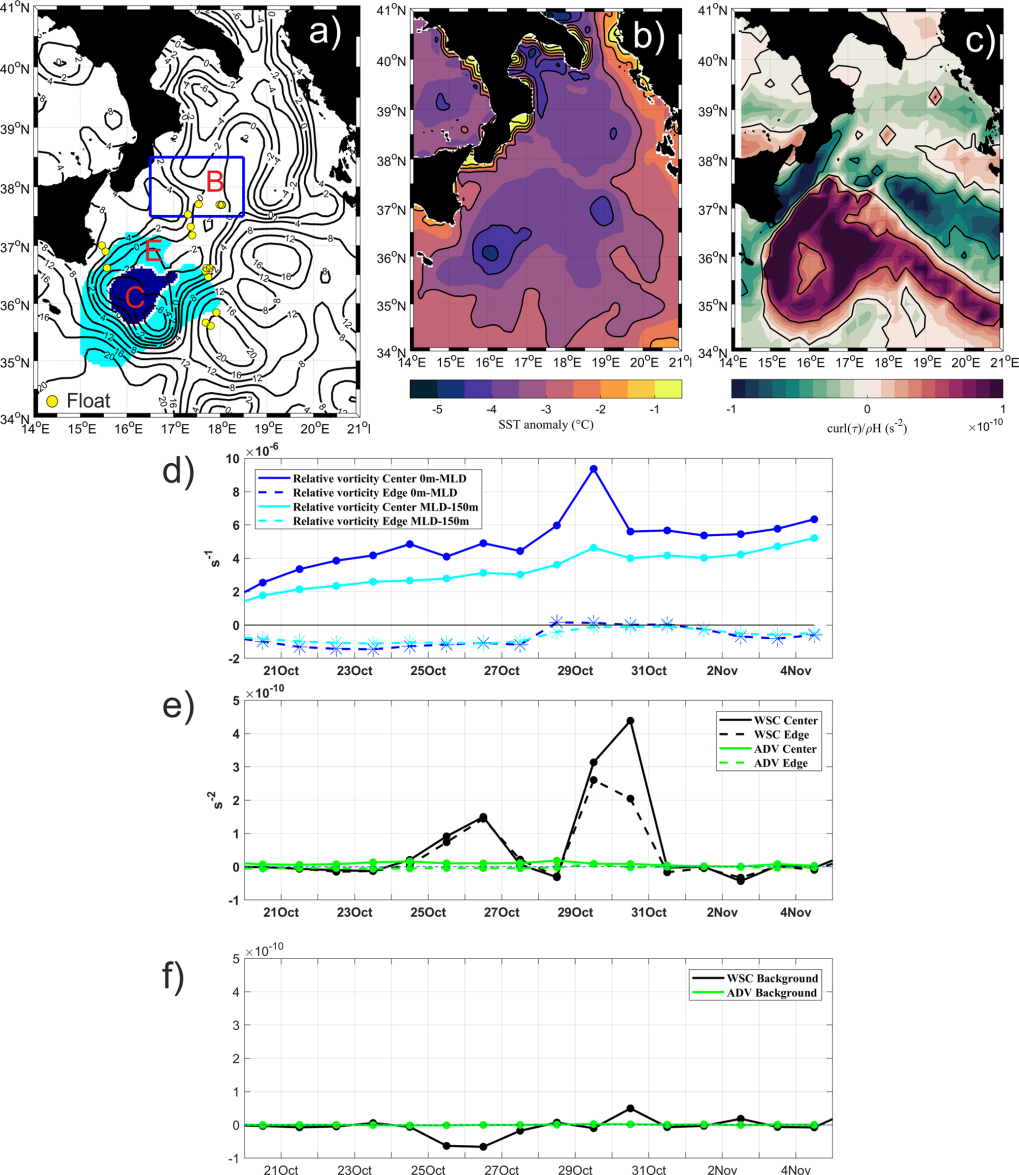
(**a**) Ionian Sea area affected by the Medicane Apollo divided in Center (C, dark blue shaded) and Edge (E, light blue shaded) regions; blue square delimits the area used as Background (B); isolines define the ADT (m) of the northern Ionian on 29 October 2021 and yellow dots show the position of the float cycles available during the event; (**b**) SST anomaly between 29 October 2021 and the mean of the first two weeks of October 2021; (**c**) wind stress curl on 29 October 2021. Time series of the **(d**) relative vorticity, (**e**) wind stress curl (WSC), and horizontal advection (ADV) terms of the vorticity equation computed in the Center and Edge and in the (**f**) Background. Positive/negative wind-stress curl is related to the increment of cyclonic/anticyclonic ocean vorticity.

In order to delimit the area most affected by the passage of Apollo (Fig. 4a), two criteria were used. The first one, (1) was based on finding the relative local negative maximum of SST anomaly computed with respect to the period before the event (1–14 October 2021, Fig. 4b), and used to identify the area with the highest Medicane-induced variability. The second criterion, (2) was based on finding the largest local positive wind-stress curl values (Fig. 4c), and used to identify a larger area around the core where the wind-induced Ekman pumping affected the vertical structure of the water column. According to (1), the area southeast of Sicily, enclosed by the isotherm of − 4 °C (Fig. 4b), was defined as the region most affected by the passage of Apollo and corresponded to the core of the cyclonic gyre encompassing the ISV and SISV (blue area in Fig. 4a; hereafter “Center”). According to (2), the area outlined by larger wind-stress curl (approximately 5 × 10^–11^ s^-2^; Fig. 4c), surrounding the “Center”, was used to delimit the spatial extent of the area impacted by Apollo and coincided with the edge of the cyclonic gyre (light blue area in Fig. 4a; hereafter “Edge”). In order to compare the temporal evolution of the physical and biogeochemical properties in the Medicane-impacted area with those not directly impacted by the system, a “Background area” was selected (blue square in Fig. 4a), by taking into account the availability of float data and avoiding the regions characterized by intense anticyclonic vortices (Fig. 3a).

The time series of the physical and biogeochemical parameters in the Center, Edge and Background areas of the Ionian Sea were analyzed and described in two layers: the surface layer, that ranges from the top of the water column to the bottom of the mixed layer (also defined as Mixed Layer Depth, MLD); and the subsurface layer, that ranges from the bottom of the mixed layer to a depth of 150 m. During the In-storm period, the relative vorticity increased suddenly both in the surface and in the subsurface layers of the Center sector (Fig. 4d). An analysis of the different terms contributing to the vorticity balance (for more details, see Method section) revealed larger contributions from horizontal advection and wind-stress curl (Fig. 4e; Figure S1). These two terms had a comparable magnitude (in the order of 10^–11^) in the Pre-storm and Post-storm periods. On the other hand, during the In-storm period, the positive wind stress curl clearly dominated the vorticity budget (order of magnitude of 10^–10^), reaching maximum values in the Center sector (Fig. 4e). These strong positive values induced a positive Ekman pumping (Table 1), and therefore a significant upward vertical velocity in the water column and, at the same time, an increment of the Ekman—layer thickness (see Ekman depth in Table 1). No differences were observed between the two terms in the Background area in the three selected periods (Fig. 4f). The other two terms of the vorticity equation, baroclinicity and tube stretching, although impacted by the Medicane passage, contributed negligibly to the total balance (Figures S1b, S1c).

**Figure 5:**
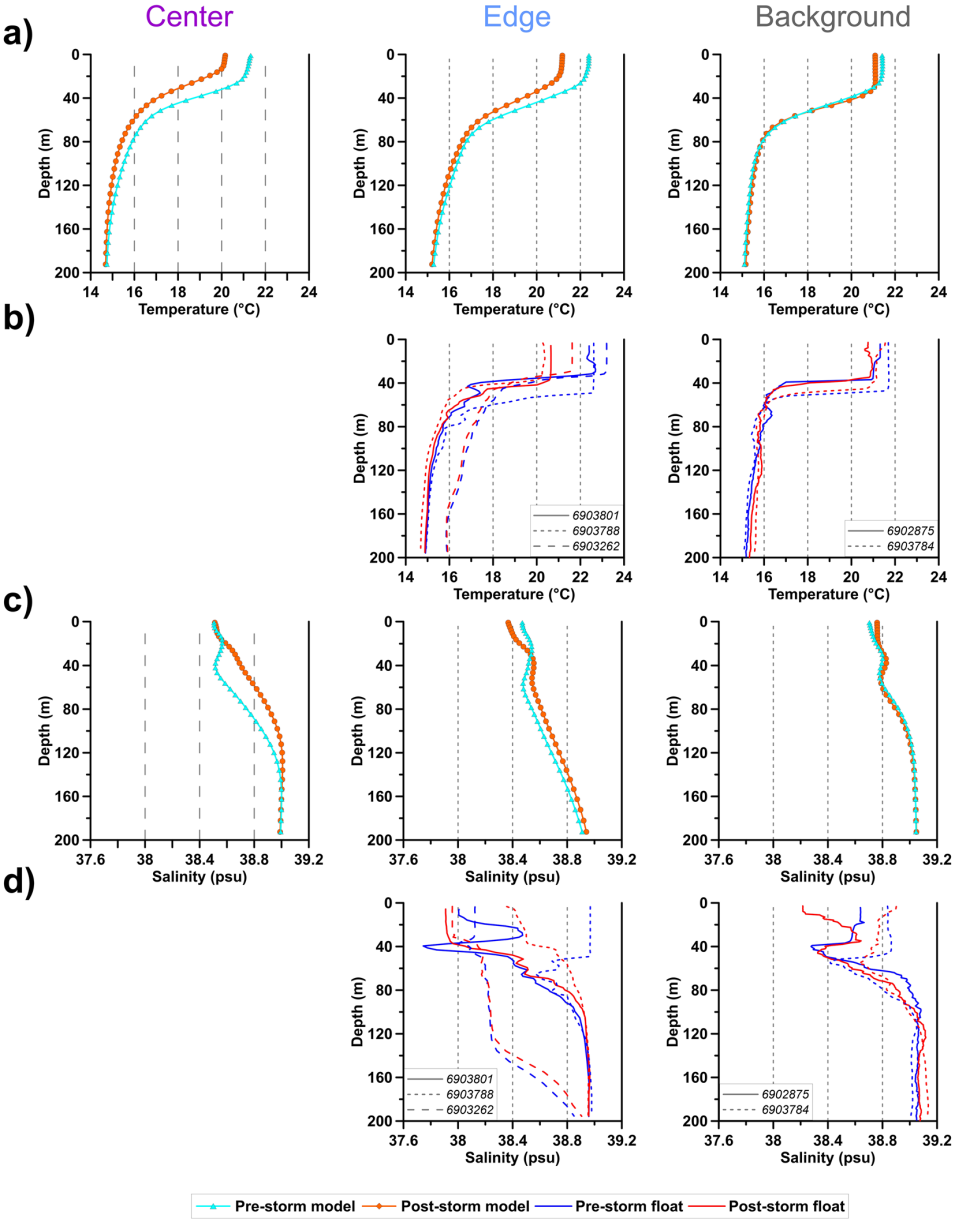
Mean temperature (**a**,**b**) and salinity (**c**,**d**) profiles derived from the physical CM model and from Argo floats for the Pre-storm and Post-storm conditions, in the Center (first column), Edge (central column) and Background (right column) sectors. The identification numbers of the Argo floats used are listed in the legend of the corresponding subplots.

Temperature dropped in the Center and Edge sectors (Fig. 5a,b) with a consequent enhancement of the vertical mixing (Table 1). Salinity values derived from CM model were almost constant in the mixed layer of the Center sector, (Fig. 5c), whereas they increased in the subsurface layer of the Center and Edge sectors (mean salinity anomaly of 0.16; Table 1), as a consequence of the upwelling that lifted the depth of the halocline favoring the upward transport of saltier water from the underlying layers (Fig. 5c). Conversely, salinity decreased in the surface layer of the Edge sector (Fig. 5c,d), because of the inflow of less salty water carried by the MIJ (Fig. 3a,c) with a contribution of intense rainfall that reached its maximum intensity over the area (Fig. 2e). CM model data were averaged in the Edge sector, while the floats, being pointwise and distributed on both east and west sides of the Edge sector (see Fig. 1), and tend to be more variable than CM model.

The float 6903788 (Table 1; Fig. 5b,d), which drifted close to the south-eastern tip of the Sicilian coast, moving from the western border toward the center of the impacted region (Fig. 1), showed the largest negative SST (∆T = − 2.01 °C), Ocean Heat Content (OHC; ∆OHC = − 0.14·10^10^ J m^−2^) and salinity anomalies (∆S = − 0.52) compared to the other floats and to the CM model. This region is characterized by intense cumulative precipitations during the storm (Fig. 2d). In the Background area, no substantial differences in temperature and salinity were observed in the water column during the Pre-storm and Post-Storm periods (Fig. 5a–d, third column).

### Biogeochemical conditions

**Figure 6:**
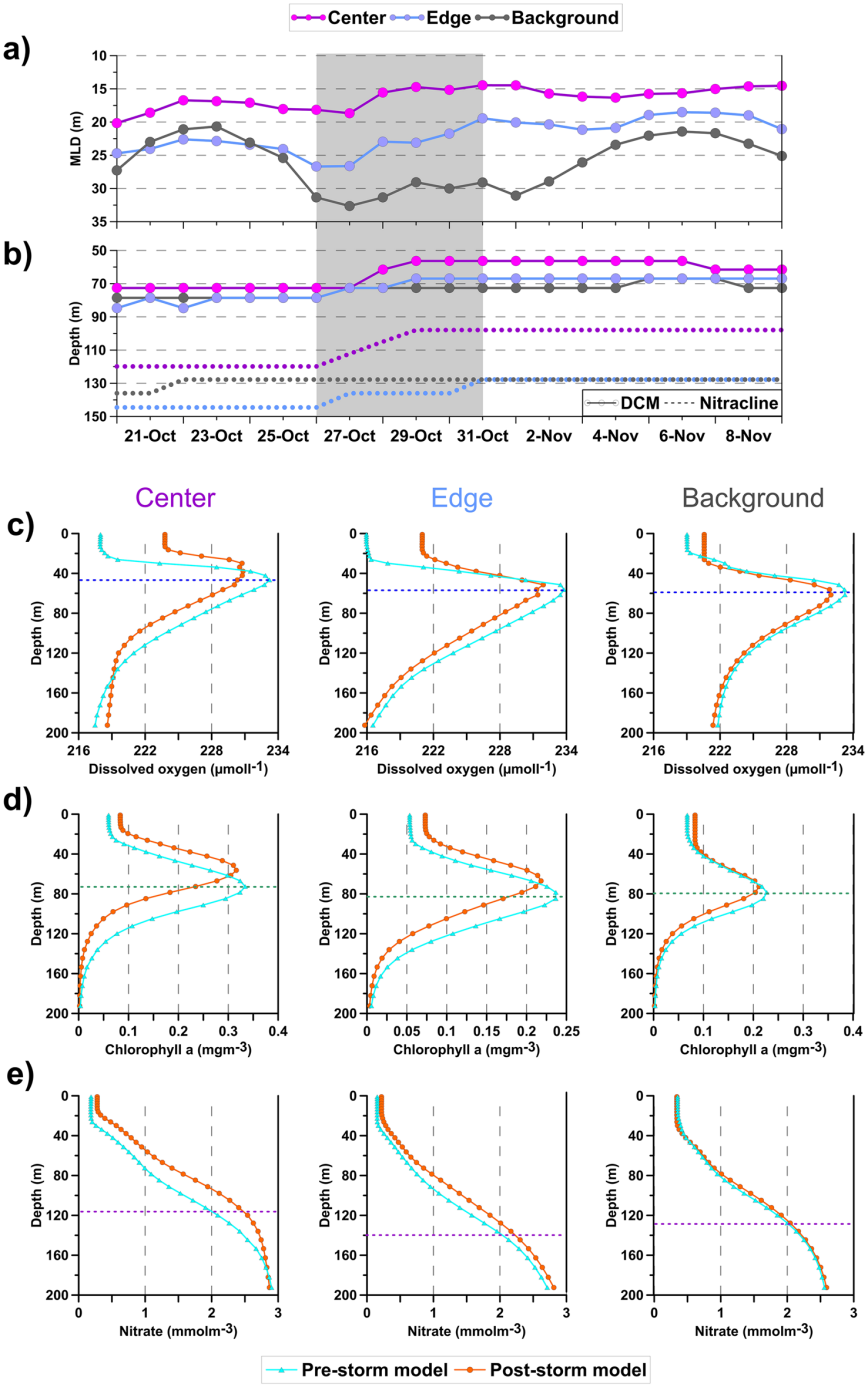
Time series of the MLD (**a**), DCM and nitracline (**b**) covering the Pre-storm, In-storm (grey shaded area) and Post-storm time periods derived from biogeochemical CM model. Mean profiles of the Dissolved Oxygen (**c**), Chlorophyll-a (**d**) and Nitrate concentration (**e**) derived from the biogeochemical CM model for the Pre-storm and Post-storm conditions, in the Center (first column), Edge (central column) and Background (right column) sectors.

Mixing events during the passage of Apollo substantially altered the vertical profiles of several biogeochemical variables in the Medicane impacted areas. The uplift of the MLD both in the Center and Edge sectors (Fig. 6a) is concomitant with a marked shoaling (higher in the Center) of the nitracline and DCM (Fig. 6b). On the other hand, in the Background sector, the observed changes were reduced. These changes persisted several days after the storm (Fig. 6a,b) showing, as expected, that the impacts of a severe weather system on the physical-biogeochemical properties have a time scale longer than the atmospheric trigger^20^.

The pre-storm period showed the typical end-of-summer condition of the Ionian Sea^50,51^ with a nitracline located at approximately 120–140 m, a DCM at about 80 m and a subsurface oxygen maximum (SOM) 30 m above the DCM (dashed lines in Fig. 6c–e). The Center sector, being also the center of a cyclonic gyre, displayed an uplifting of the isosurfaces with respect to the Edge (i.e., a dome shape). Indeed, nitracline, DCM and SOM depths of the Center were about 5–10 m shallower than the Edge and Background sectors.

Apollo impacted ocean biogeochemistry through two different mechanisms: (1) By uplifting the isosurfaces of the cyclonic dome shape (i.e., upwelling driven by Ekman pump), the vertical profiles of biogeochemical variables were shoaled. (2) By enhancing the vertical mixing, the surface layer was enriched in nutrients that stimulated phytoplankton growth (i.e., increase in primary production, not shown) and increased chlorophyll-a concentration at the surface. Additionally, while the decrease in water temperature increased oxygen solubility, the increase in wind speed boosted air-sea transfer and surface mixing. As a result, the surface layer, was enriched in oxygen of about 6 mmol m^−3^ in just a few days (Fig. 6c). While the cyclonic dome was uplifted almost uniformly both the Center and Edge areas, the temperature drops and increase in surface mixing supported slightly more an increase of oxygen concentration at the surface in the Center area. The Background sector reported negligible changes in nutrient and chlorophyll-a profiles, and only a small increase of oxygen concentration at surface (~ 1 mmol m^−3^).

## Discussion

Nowadays, extreme weather systems such as Medicanes are studied with increasing interest, as they can have potentially damaging natural and socioeconomic consequences. Recent simulations projected a decrease in their frequency in the future, but also an increase in their intensity and duration^33,52,53^. TCs intensification and predictability are strictly linked to the preexisting upper-ocean conditions^6,54,55^. Satellite data are an important source of information but have some limitations; e.g. they provide information only at sea surface and capture only partially the SST drop and the surface chlorophyll-a increase associated with the strong vertical mixing^21^, because the intense rainfall and the cloud cover during the event contaminate satellite observations. Modeled data reconstruct the 3D physical and biogeochemical variables during the passage of severe weather systems, but they can be affected by large uncertainties^56^. In this context, autonomous instruments in the Mediterranean allow us to study the effect of Medicanes in real-time and provide critical in-situ measurements at and beneath the ocean surface useful to initialize numerical weather prediction models^54^.

In this work, we combined autonomous oceanographic instruments with satellite and model products to describe the impacts of an extreme weather system on a cyclonic gyre in the Mediterranean Sea. Recent studies focused on the responses of ocean eddies to TCs in several areas of the global ocean, revealing a large variability of their impacts, depending on the complex dynamics of the systems and on the local factors^57–61^. TCs interaction with cyclonic eddies can further strengthen the TC-induced surface cooling^59,60^, through the combined effects of upwelling and wind-driven mixing, weakening in turn the strength of the atmospheric system through negative feedback^55,57,62,63^. Medicane Apollo seems to be an example of such “gyre negative modulation effect” on the atmospheric system. The system showed its maximum intensity on October, 29^th^ 2021 (Fig. 2b,c), when it impacted on the cyclonic gyre (Fig. 3a), and then its intensity was reduced rapidly during the hours in which it remained over the gyre (Figs. 2a,b, 3a). Although there are no detailed meteorological studies on Apollo yet, it is reasonable to think that it transitioned from an extratropical to a tropical-like cyclone just after 28 October at 18:00.

Oceanic eddies are structures highly variable in space and time, frequently changing their position and lifetime. The cyclonic vortex impacted by Apollo is not an eddy but a Mediterranean gyre, a permanent structure located in a specific geographical area and well defined from a dynamical point of view^64,65^. To our knowledge, no earlier work has addressed so far the impact of an extreme weather system on such a permanent structure of the Mediterranean Sea. Bouin et al.^36^ described the impact of the cyclone Qendresa on a cyclonic structure (Adventure Bank Vortex—ABV) of the Sicily Channel in 2014. However, their condition was very different from the one addressed in this paper, as cyclone Qendresa impacted the ABV during its developmental phase (i.e., when it was still classified as an extratropical cyclone) while during its transition phase (i.e., when it was classified as a Medicane), it was located east of Malta, along the eastern Sicily coast, far from the ABV. A further difference is that in^36^ does not use any biogeochemical or in-situ data.

**Figure 7:**
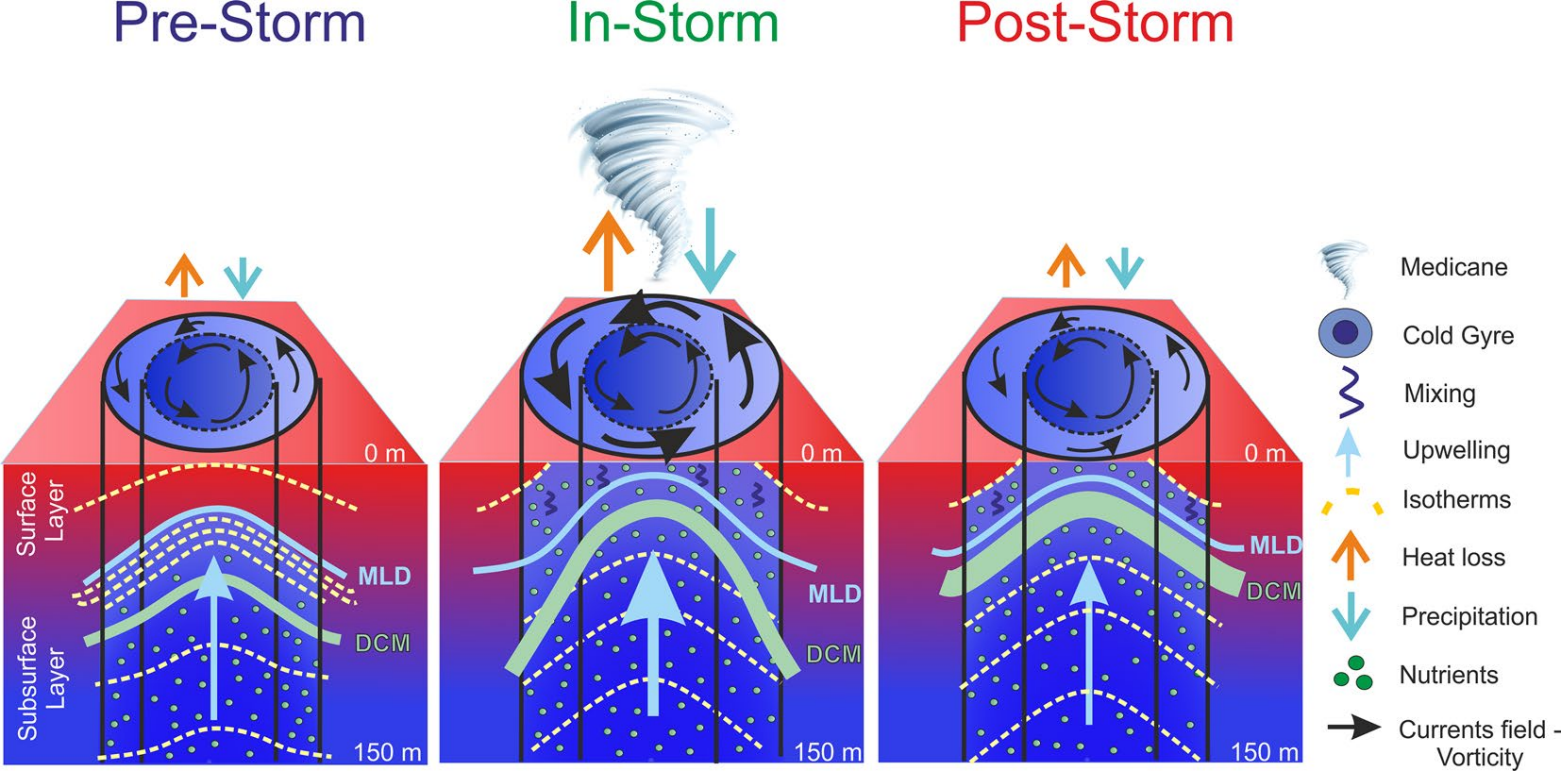
Schematic representation of the effects of Medicane Apollo on the Ionian Sea cyclonic gyre. The colors range from red to blue representing higher and lower temperatures, respectively.

The atmosphere/ocean interaction described in this work generated different ocean responses than expected, providing a useful case study for better predicted the behavior of extreme weather systems in the future. At the end of October 2021, before the passage of Medicane Apollo, the cyclonic gyre located southeast of the Sicily coast was outlined by a warm, stratified surface layer, ranging from 0 m to the MLD (~ 20 m and ~ 25 m in the Center and Edge sectors, respectively; Fig. 6a), and by a colder subsurface layer (ranging from the MLD to 150 m depth) affected by upwelling (Fig. 7, Pre-storm; Figure S2, upper left panel). The DCM was located at about 80 m depth (Figure S2, lower left panel); nutrient concentration increased with depth in the subsurface layer, and the nitracline was located at about 120 m depth (Fig. 6b,e; Figure S2 middle left panel). The core of the cyclonic gyre was characterized by the typical upward doming of the isosurfaces (Fig. 7, Pre-storm; Figure S2, left column). During the passage of the storm (Fig. 7, In-storm condition), heat losses and precipitations increased, with maxima values along the edge of the cyclonic gyre (Fig. 2d,e). The mechanical energy transferred from the atmosphere to the sea generated wind-driven upwelling (Fig. 4e), induced vertical mixing in the surface layer and strengthened the cyclonic current field (Figs. 2c, 4d). The strong upwelling in the subsurface layer raised the MLD and the nitracline, with a consequent uplifting of DCM of ~ 20 m (Fig. 7, In-storm condition; Figure S2 middle column). The uplift of the water column was associated with the squeezing of the surface layer (0-MLD m), confirmed by the strong negative contribution of the tube stretching term to the vorticity balance on October, 29^th^ (though one order of magnitude smaller than the dominant wind-stress curl term, Figs. 4e and S1c), and by the widening of the area affected by temperatures below 21 °C compared to the Pre-Storm condition (Figure S2, upper panels). After the storm (Fig. 7; Post-storm condition), initial conditions were re-established in terms of heat losses, freshwater fluxes, wind-stress and subsurface upwelling. The water column, however, was more mixed, colder, and richer in chlorophyll-a and nutrients in the photic zone (Figure S2, right column).

This analysis confirms that pre-existing ocean conditions drive the physical and biogeochemical response of the marine environment to the passage of extreme weather systems. In particular, the presence of a cyclonic gyre along Apollo's trajectory leads to a different physical response compared to previously documented Medicanes. The deepening of the MLD, described by^37^ as an essential ingredient of the impact of Medicane Zorbas on the Ionian Sea (27 September 2018–2 October 2018), was not observed in the case of Apollo. On the contrary, the response of the cyclonic gyre further reduced the already shallow MLD (Fig. 6a). The lack of deepening of the MLD, related to the strengthening of upwelling in the subsurface layer, resulted in the absence of the “heat pump” effect (cooling/warming at the sea surface/subsurface layer), with a net cooling in the first 150 m of the water column (Fig. 5a,b). This result agrees with a recent review proposed by^8^, which describes a reduction of the subsurface warming under combined mixing plus upwelling conditions, with potential absence of this warming in the case of very intense upwelling (see Fig. 2 of^8^). The impact of the Medicane Ianos (14–21 September 2020) on the Ionian Sea increased substantially the significant wave height (up to 6 m) and of the sea level along its track^35^. On the contrary, the presence of a cold gyre along the Apollo track mitigated the impact on significant wave height and on the sea level (Table 1). The largest significant wave height of ~ 3.2 m was observed during the In-storm period, with an increment of ~ 2 m compared to the Pre-storm period (Table 1).

The decrease of salinity in the surface layer of the area characterized by higher rainfall, observed with Medicane Apollo (Figs. 2e, 5), was previously described by the simulations of the Medicane “Qendresa” (Sicily Channel, November 2014), performed by^36^. These authors found that salinity controls the change in density with a corresponding decrease at the surface and large shoaling of the MLD.

For Apollo, the combined effect of vertical mixing and upwelling resulted in a shoaling of the DCM and of the nitracline (Fig. 6b). Dissolved oxygen and chlorophyll-a concentrations increased at the surface, due to enhanced solubility and productivity induced by cooling and strong vertical mixing, and decrease in the subsurface waters, due to the upwelling of less oxygenated and poorer in chlorophyll-a deep waters (Fig. 6). These results are in agreement with those expected in the literature also concerning the coupling between the physical and biogeochemical dynamics^20,21,66^, although they are triggered by different dynamical forcings. According to the scheme proposed by^21^, the increase of chlorophyll-a concentration in the surface layer is related to MLD deepening, and to the consequent enrichment of the surface layer with DCM waters through mixing. In the case of Medicane Apollo, the strong upwelling in the interior of the cyclonic gyre uplift the entire water column with a consequent shoaling of the biogeochemical properties.

Our analysis demonstrates how a coordinated, multi-platform observing system integrated to an operational model in the Mediterranean Sea, dedicated to monitoring ocean and atmospheric parameters would be beneficial to improve our knowledge of the complex processes that may be linked to the Medicanes intensification, their impacts and/or to other extreme events. It is desirable that the scientific community and policymakers will soon get organized to coordinate the capability of atmospheric forecasts (e.g., ECMWF), marine operational and monitoring systems such as those provided by the Copernicus Marine Service^66^ and supporting advanced observing synergistic networks such as Argo^44^ for this purpose.

## Methods

The trajectory of Apollo was reconstructed by applying an objective procedure to the ERA5, 6-hourly, gridded (0.25° × 0.25°) Mean Sea Level Pressure (MSLP) fields for the period October–November 2021. The procedure, which is described in^47,67^, is based on the partition of the MSLP field at a certain time T_n_ in a number of depressions by the identification of sets of steepest paths leading to the same MSLP minimum. In this procedure, each point of the MSLP field is connected to the lowest of the 8 nearest-neighbor points and this step is repeated until a minimum is reached. Then all the points crossed by a path leading to the same minimum are assigned to the same cyclone. Finally, the track of each system is built by joining the locations of the same cyclone center in successive time steps. The final result of this automatic scheme is a list of cyclones with the associated position as a function of time in coordinates of longitude/latitude and the temporal evolution of variables such as the MSLP minimum, Laplacian, Gradient, Depth, Size and Position of the maximum of Pressure Gradient and Laplacian within each depression.

The cyclone depth is estimated as the difference between the value of MSLP at the center of the system and the average MSLP in a certain distance from the center of the system itself (background field; see^47^ for more details).

The surface drifter moving along the eastern Sicilian coast during the event (IMEI 300234067977120) was equipped with a barometer and was programmed to transmit every hour. Data were processed using the OGS standard procedures^45^. Measurements of the water-following capabilities of this drifter design have shown that, when the drogue is attached, it follows the water to within ± 1 cm s^−1^ in 10 m s^−1^ winds^68^.

Air-sea interactions properties were evaluated using the daily radiative fluxes (shortwave and longwave radiations, respectively Q_SW_ and Q_LW_) turbulent heat fluxes (latent and sensible flux, respectively Q_lat_ and Q_sen_) and precipitation (P), downloaded from ERA5 (spatial resolution of 0.25° × 0.25°). Surface net heat flux (Q_net_) is a combination of the radiative and turbulent fluxes obtained as:

$${\text{Q}}_{{{\text{net}}}} = {\text{ Q}}_{{{\text{SW}}}} - {\text{ Q}}_{{{\text{LW}}}} - {\text{ Q}}_{{{\text{lat}}}} - {\text{ Q}}_{{{\text{sen}}}}$$

Cumulative Q_net_ and P in the Ionian Sea were estimated in the Pre-storm, In-storm and Post-storm periods.

The daily Absolute Dynamic Topography (ADT) and correspondent Absolute Geostrophic Velocities (AGV) derived from altimeter and distributed by Copernicus Marine Service^69^ were used to describe the surface currents and circulation features on October, 29^th^ 2021. The ADT was obtained by adding the sea level anomaly to the 20-years synthetic mean estimated by^70^ over the 1993–2012 period.

Daily surface temperature, salinity, currents and MLD fields (0–150 m depth) were derived from the Copernicus Marine Service Analysis and Forecast physical product^71^ (spatial resolution of 0.042° × 0.042), whereas nitrate, chlorophyll-a and dissolved oxygen were derived from Copernicus Marine Service Analysis and Forecast biogeochemical product^72,73^ (spatial resolution of 0.042° × 0.042). The physical and biogeochemical Copernicus Mediterranean Sea (CM) model consists of the NEMO-WW3 and BFM models including data assimilation of SLA, satellite chlorophyll-a and profiles of temperature, salinity, chlorophyll-a and nitrate^74–76^. These products were used to define the mean vertical profiles of each variable and the time series of the MLD, DCM, SOM and nitracline in the Center, Edge and Background sectors, during the Pre-storm and Post-storm time periods. DCM and SOM are defined as the depth at which the maximum concentration of chlorophyll-a and oxygen occurs within the 0–200 m layer. Nitracline is defined as the depth at which the concentration of Nitrate reaches the values of 2 mmol m^3^ starting from the surface.

The Argo float data^43^ selected for this work were derived from five platforms, three of them located in the Edge sector and the other two located in the Background sector, for a total of 19 cycles (Fig. 1; Table 1). Vertical temperature and salinity profiles in the Pre-storm and Post-storm periods were retrieved and qualitatively compared with the profiles derived by the physical CM model (see Table 1). The OHC for the CM model and Argo float profiles was estimated following the method of^77^. Mean values of Chl derived from the biogeochemical CM model in the surface layer (0-MLD m) were compared with the satellite ocean colour data^78^ distributed by Copernicus Marine Service. According to the availability of satellite data in the Medicane impacted area, maps of 21 October, 30 October, and 2 November 2021, at 00:00 UTC, were selected as representative of the Pre-storm, In-storm, and Post-storm period, respectively (see Table 1).

The relative vorticity field (ζ) of the velocities derived from the physical CM model (*V*) in the surface (0-MLD m) and subsurface (MLD-150 m) layers was evaluated as the vertical component of the horizontal current velocity field curl:

$$\zeta = \frac{\partial v}{{\partial x}} - \frac{\partial u}{{\partial y}};$$

where *u* and *v* are the zonal and meridional components of velocity *V*, respectively. Daily current vorticity fields were spatially averaged in the Center, Edge and Background regions obtaining a time series of the period spanning the Medicane event. The vorticity equation was analyzed in order to evaluate the relative importance of various sources of current vorticity. Neglecting the bottom stress, the resulting vorticity equation is:

$$\frac{\partial \zeta }{{\partial t}} = - V\cdot\nabla \zeta + \frac{\nabla p \times \nabla \rho }{{\rho_{0}^{2} }} + f\left( {\frac{\partial w}{{\partial z}}} \right) + \frac{1}{{\rho_{0} D}}\left[ {curl\tau } \right]_{z} ;$$

where the first term ($- V\cdot\nabla \zeta$) is the advection from neighboring areas; the second term is the baroclinicity, with p and $\rho_{0}$ the pressure and the density (1025 kg m^−3^) of seawater, respectively; the third term is the tube stretching, with *f* the Coriolis parameter and *w* the vertical component of the velocity; the forth term describes the wind-driven contribution to the vorticity field, with *D* the thickness of the upper layer (0-MLD m depth) and $\left[ {curl\tau } \right]_{z}$ the vertical component of the wind-stress curl:

$$\left[ {curl\tau } \right]_{z} = \frac{{\partial \tau_{y} }}{\partial x} - \frac{{\partial \tau_{x} }}{\partial y};\quad \left( {\tau_{x} ,\tau_{y} } \right) = \rho C_{D} \left( {u_{w} ,v_{w} } \right)V_{10} ;$$

where $\left( {\tau_{x} ,\tau_{y} } \right)$ are the wind-stress components, $\rho$ (1.22 kg m^−3^) is the density of air, $\left( {u_{w} ,v_{w} } \right)$ and $V_{10}$ are the components and the magnitude of the wind speed at 10 m, respectively, and $C_{D}$ is the drag coefficient already used in the Mediterranean Sea by^48,79^.

The order of magnitude of the baroclinic term is estimated following^80^, as $O\left( {\frac{{f^{2} V^{2} }}{gD}} \right)$, where *g* is the gravitational acceleration. The contribution of the tube stretching term to the vorticity balance is estimated using the continuity equation to derive $\left( {\frac{\partial w}{{\partial z}}} \right)$.

### Supplementary Information

Supplementary Figures.

## Data Availability

Publicly available datasets were analyzed in this study. These data can be found in: https://www.ecmwf.int/en/forecasts/datasets/reanalysis-datasets/era5; https://www.coriolis.eu.org/Observing-the-Ocean/ARGO; 10.25423/CMCC/MEDSEA_ANALYSISFORECAST_PHY_006_013_EAS6; 10.25423/cmcc/medsea_analysisforecast_bgc_006_014_medbfm3; 10.48670/moi-00141; 10.48670/moi-00299.
