# Author Correction: A case study of impacts of an extreme weather system on the Mediterranean Sea circulation features: Medicane Apollo (2021)

**DOI:** 10.1038/s41598-024-64630-3

**Published:** 2024-06-14

**Authors:** Milena Menna, Riccardo Martellucci, Marco Reale, Gianpiero Cossarini, Stefano Salon, Giulio Notarstefano, Elena Mauri, Pierre-Marie Poulain, Antonella Gallo, Cosimo Solidoro

**Affiliations:** Oceanographic Section, National Institute of Oceanography and Applied Geophysics - OGS, 34010 Sgonico, TS Italy

Correction to: *Scientific Reports* 10.1038/s41598-023-29942-w, published online 08 March 2023

The original version of this article contained an error in Reference 73, which was incorrectly given as:

Salon, S. *et al.* Marine ecosystem forecasts: Skill performance of the CMEMS Mediterranean sea model system. *Ocean Sci. Discuss.* **1**, 35. 10.5194/os-2018-145 (2019).

The correct reference is listed below:

Salon, S. *et al.* Novel metrics based on biogeochemical argo data to improve the model uncertainty evaluation of the CMEMS Mediterranean marine ecosystem forecasts. *Ocean Sci.* **15**, 997–1022. 10.5194/os-15-997-2019 (2019).

The original article has been corrected.

