# Supplementary material for: A case study of impacts of an extreme weather system on the Mediterranean Sea circulation features: Medicane Apollo (2021)
Source: Sci Rep. 2023 Mar 8;13:3870. doi: 10.1038/s41598-023-29942-w (PMC9995500; doi:10.1038/s41598-023-29942-w)
Supplement: Supplementary file 1 — Supplementary Figures. [file 41598_2023_29942_MOESM1_ESM.docx]

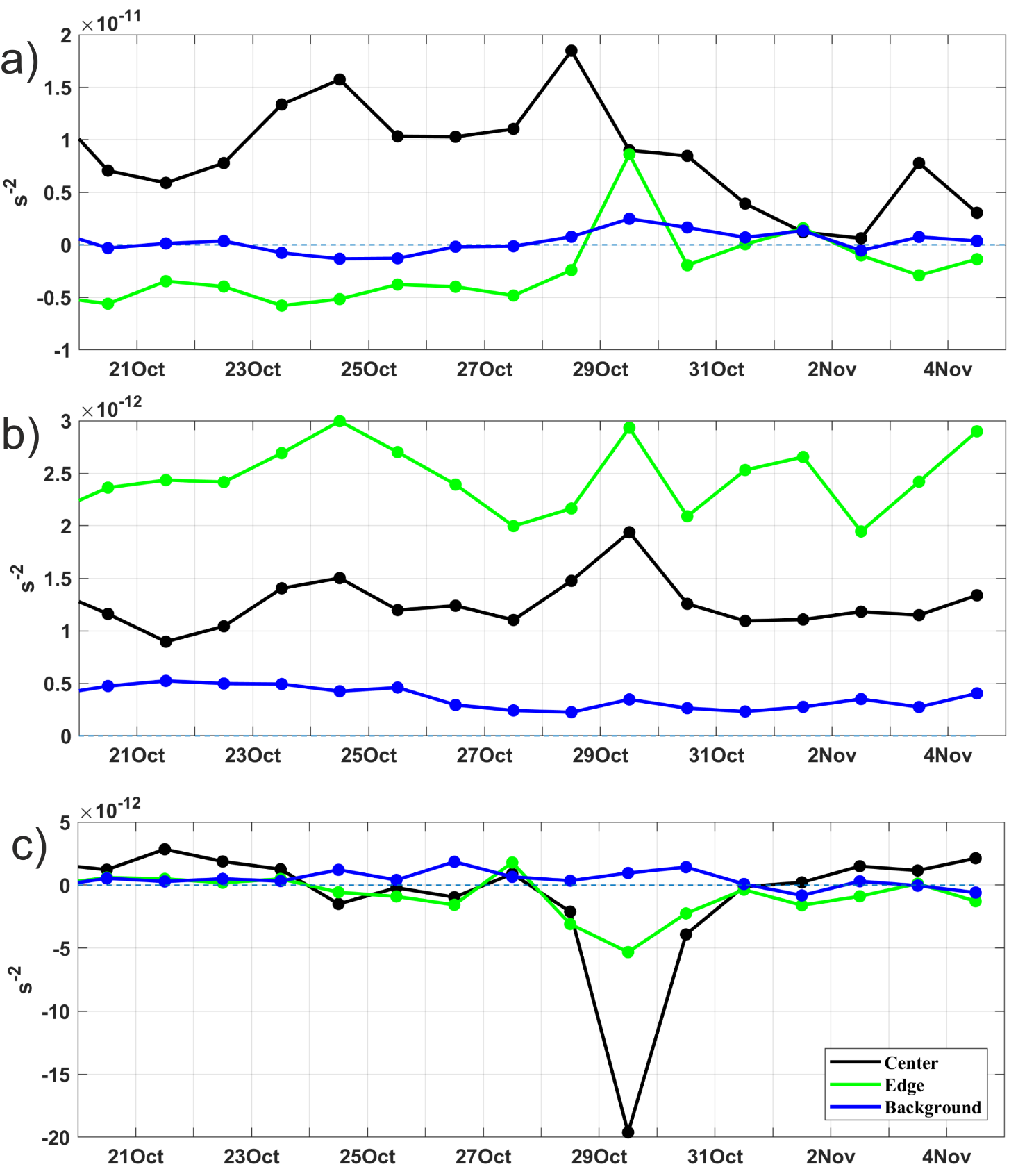


Figure S1. Time series of the a) horizontal advection, b) baroclinic and c) tube stretching terms of the vorticity equation (see Method Section) computed in the Center, Edge and Background sectors.


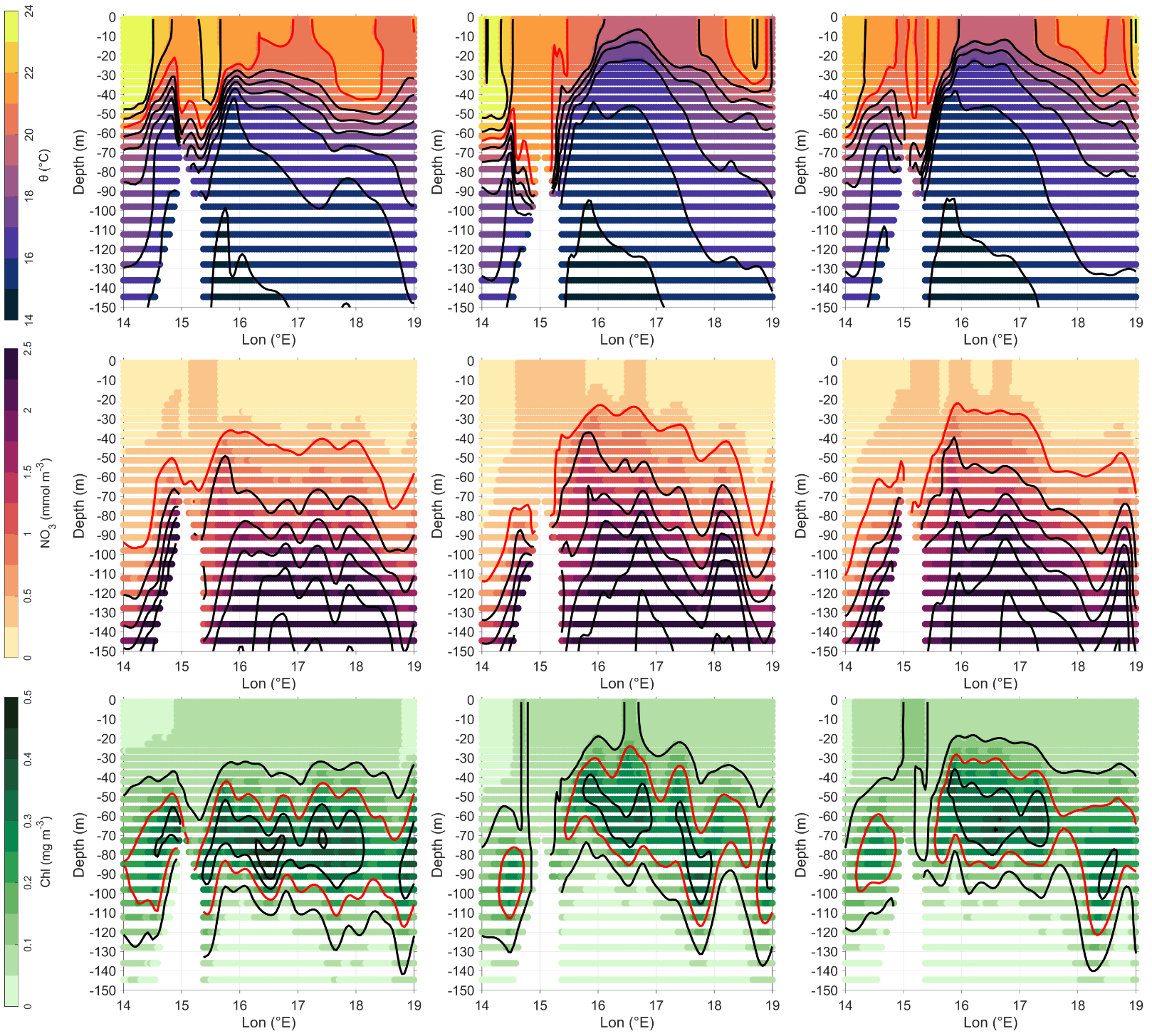


Figure S2. Vertical distribution of the potential temperature (θ, upper row), nitrates (NO_3_, mid row) and chlorophyll-a (Chl, lower row) in the Pre-Storm (left column; October 24^th^, 2021), In-storm (middle column, October 30^th^, 2021) and Post-Storm (right column; November 3^rd^, 2021) periods along a longitudinal transect located at 36.5°N. The vertical extension of the cyclonic gyre is characterized by the typical upward doming of the isosurfaces in the region between 15.2°E and 18°E; the downward doming of the isosurfaces between 14.8°E and 15°E corresponds to the thermohaline front between the AIS-MIJ and the cyclonic gyre. The surface water volume located within the cyclonic gyre and contoured by the 21°C isotherm (depicted in red in the upper row subplots) describes the widening of the surface area affected by cold temperatures during the In-storm period compared to the Pre-storm condition. The isolines of 0.5 mmol·m^-3^ and of 0.2 mg·m^-3^ in the NO_3_ and Chl-a concentration, respectively, are marked in red (middle and lower rows subplots), in order to highlight the uplift of the water column during the In-storm period compared to the Pre-Storm, and the persistence of this condition also in the Post-Storm period.
